# Evaluation of health status and its predictor among university staff in Nigeria

**DOI:** 10.1186/s12872-018-0918-x

**Published:** 2018-09-20

**Authors:** Elizabeth M. Joseph-Shehu, Busisiwe P. Ncama

**Affiliations:** 1 School of Nursing and Public Health, Postgraduate Office, Ground Floor, George Campbell Building, Howard College Campus, University of KwaZulu-Natal, Durban, South Africa; 2 Department of Nursing Science, Faculty of Health Sciences, National Open University of Nigeria, Abuja, Nigeria

**Keywords:** Health status, Noncommunicable diseases, Blood pressure, Waist-hip ratio, Body mass index, Random blood sugar, University staff, Nigeria

## Abstract

**Background:**

Hypertension, diabetes mellitus and obesity share some characteristics in relation to diagnosis, management, and prevention. Overweight, obesity and waist-hip ratio (WHR) are associated with increased risk for development of diabetes and hypertension. Surveillance and regular screening exercises are essential in control and prevention of overweight, obesity, diabetes and hypertension. There is limited literature that reported on these health status parameters among university staff in low- and middle-income countries such as Nigeria. It is currently unclear whether Nigerian have a high or low proportion of metabolic risk factors. Therefore, the study aims to examine health status parameters and their predictors among university staff in Nigeria.

**Methods:**

The study used a cross-sectional descriptive design. Data were collected from 280 university staff in Nigeria. A self-administered questionnaire with sections for sociodemographic data and physical assessment was used to gather information from the participants. Data were analysed using the Statistical Package for Social Science (IBM-SPSS version 25). Univariable and multivariable logistic regression was conducted to explore the association between predictors and health status parameters of the participants.

**Result:**

The response rate was 87.5%. University staff had mean systolic blood pressure of 132.04 mmHg ± 19.20 mmHg, diastolic blood pressure of 78.11 mmHg ± 10.81 mmHg, body mass index of 27.74 ± 5.22, waist-hip ratio of 0.88 ± 0.68 and random blood sugar of 98.65 ± 21.30 mg/dL. Predictors of high blood pressure were age (adjusted odds ratio [aOR] = 1.10, CI 95%: [1.05–1.14]) and gender (aOR = 0.5, CI 95%: [0.8–0.9]) and predictors of body mass index were gender (aOR = 2.3, CI 95%: [1.3–4.2]) and religion (aOR = 0.3, CI 95%: [0.2–0.7]). Gender and age had statistically significant association with waist-hip ratio and random blood sugar respectively.

**Conclusion:**

The prevalence rates of high blood pressure and random blood sugar; overweight, obesity and risk WHR are on the increase compared to previous studies. Lifestyle modification, organized and explicit health campaigns coupled with regular screening and surveillance will contribute to the prevention and control of noncommunicable diseases.

**Electronic supplementary material:**

The online version of this article (10.1186/s12872-018-0918-x) contains supplementary material, which is available to authorized users.

## Background

Health status is defined in relation to blood pressure, body mass index (BMI), waist-hip ratio (WHR) and random blood sugar (RBS). These health status parameters were used to assessed cardiometabolic disorders which include disorders in glucose regulation, central obesity, dyslipidaemia and hypertension [1]. These cardiometabolic disorders are risk factors for the development of noncommunicable diseases (NCDs) such as cardiovascular diseases and diabetes.

The burden of noncommunicable diseases is well documented in low- and middle-income countries (LMICs) [2–4]. Eighty per cent of premature deaths associated with NCDs occur in LMICs, and they usually affect ages between 30 and 69 years [5–7]. Statistics show that there is an increase in global deaths associated with NCDs annually. In 2005, 60.3% deaths annually were attributed to NCDs [8], and currently the figure has risen to 70% [5, 7]. NCDs are no longer diseases of the affluent, as LMICs have the highest mortality and morbidity associated with the disease [9]. The impact of NCDs is not confined to quality of life of the affected individual alone; NCDs also affect the families and the national economy [9].

Cardiovascular diseases (CVDs) alone have a global annual death rate of 17.7 million (44.3%) and the figure for diabetes mellitus is about 4.0% [5]. Hypertension, diabetes mellitus and obesity share some characteristics in relation to diagnosis, management, and prevention [10, 11]. Waist-hip ratio (WHR) is an indicator that complements BMI measurement to identify individuals at risk of obesity-related morbidity [12]. Overweight, obesity and WHR [12] are associated with increased risk for development of diabetes, hypertension [13–15] and ischemic heart diseases [16]. BMI is significantly associated with manifestation of angina, myocardial infarction, heart failure and sudden death in both males and females [17]. Hypertension, diabetes and hyperlipidaemia are linked with coronary heart disease that have high mortality and morbidity globally [18]. Hence they are of public health concern globally [19]. One in three adults globally have raised blood pressure which is the cause of about 50% of all deaths from stroke and other heart diseases; one in 10 adults have diabetes mellitus [19] and one in four women are overweight [20].

The prevalence rate of diabetes in Africa varies between 1 to 3% and 5% to 6% among rural and urban dwellers respectively [21]. According to the International Diabetic Federation, Nigeria has the highest number of people living with diabetes and impaired fasting glucose in Africa [19]. Literature showed that by 2030, development of diabetes in developing countries will be between 45 and 64 years [11, 20, 22]. Obesity increases the prevalence rate of type 2 diabetes [20, 21], and its prevention is critical to reducing the onset of type 2 diabetes [19]. Most deaths related to CVDs occur in the working age range of 35 to 64 years [11] when the individual is still very productive. Hypertension and diabetes are usually co-existed [19].

Overweight and obesity are on the increase in LMICs [23] and will soon surpass their prevalence in the developed countries [4]. Among university staff in Nigeria there is a continued increase in the prevalence of obesity, which may be attributed to the nature of their work [24]. Development of abdominal fat occurs among both academic and non-academic staff, indicating growth of central abdominal fat [24] that increases the risk for metabolic and cardiovascular disorders [25]. World Health Organization (WHO) reports have documented that a 12-year follow-up of middle-aged men with high WHR was associated with increased risk of myocardial infarction, stroke, premature deaths and insulin resistance [12].

High blood pressure is a risk factor for stroke, ischaemic heart diseases, heart failure and chronic kidney diseases [26]. Commonly referred to as the silent killer [27], it is liable to remain untreated [28] since most people are not aware of the symptoms or warning signs. Untreated hypertension may lead to coronary heart diseases or congestive heart failure, which account for 50% of all deaths associated with hypertension; 33% of untreated hypertension may lead to stroke, and 10–15% will result in renal failures [26]. Prevalence rates for hypertension in Nigeria, as reported in various studies [6, 7, 19, 26], differ from one zone to another.

Overweight and obesity increase the rates of premature mortality and morbidity from CVDs and worsen the prognosis of type 2 diabetes, chronic back pain and osteoarthritis [23]. CVDs and diabetes mellitus account for 30% of global mortality, and 80% of these deaths were recorded in LMICs [29]. Expedient intervention is called for, especially among university staff [24], in response to increase in the burden of CVDs [3] and steady increase in prevalence rates for hypertension [26, 28] and diabetes [19] in Nigeria – coupled with the link for the latter two with overweight and obesity [20, 23, 30].

There is a paucity of data on health status that considers the four parameters that are important in the development of CVDs among university staff in Nigeria. Furthermore, there is limited literature that reported on blood pressure, BMI, WHR RBS among university staff [31, 32] in LMICs such as Nigeria. It is unclear whether Nigerian have a high or low proportion of metabolic risk factors. Hence, it is essential to examine the health status of this population before developing an intervention to prevent and control NCDs among them. The aim of this study is to examine the health status and its predictors among university staff in Nigeria.

## Methods

**Fig. 1:**
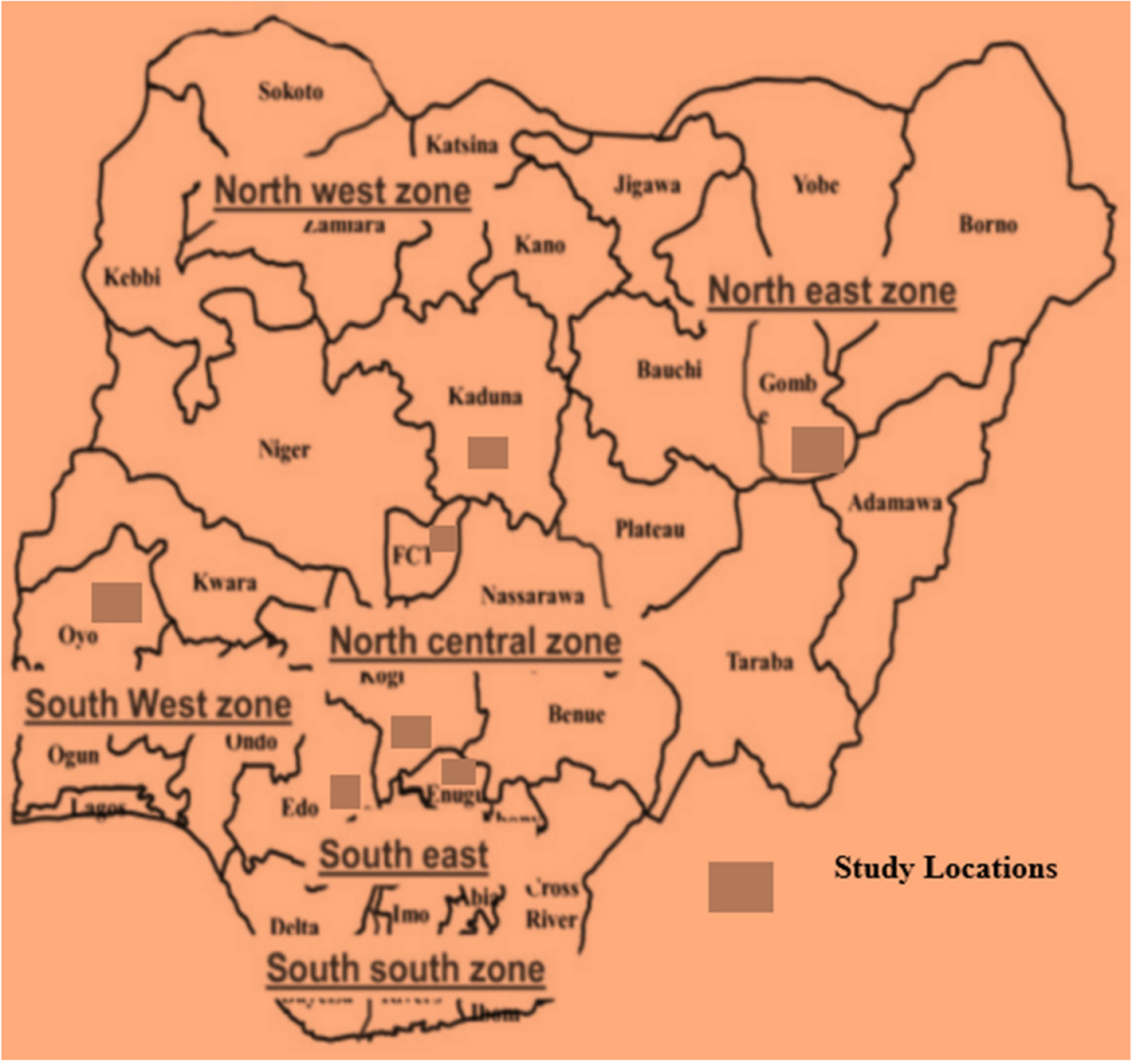
Map of Nigeria showing the six-geopolitical zones and study locations retrieved from Wordofmonk.wordpress.com on February 2016

This study is a cross-sectional, descriptive study design that was conducted among university staff in Nigeria between October and December 2016. The selected university is the largest in term of spread, and student population. The university has a headquarters and over 70 satellite campuses with 2657 staff across the country as of 2016. The study settings were spread across the six geopolitical zones in Nigeria; Fig. 1 shows the study locations. One state was randomly selected (ballot method) in each geographical zone and purposive selection was used in choosing the institution headquarters and the study centres.

G*Power 3 [33] was used in determine a figure of 231 for the study sample size. The sample size was increased by 15% to make it 266 to allow for a non-parametric equivalent should the proposed parametric test not be valid. The study participants were drawn from the major study centers (usually one per state) in each of the selected states. A convenience sampling technique was used to select participants for the study. Data were collected from 141 participants in the selected study centers and 139 from headquarters who were willing to be part of the study.

A self-administered questionnaire (Additional file 1) that consisted of two sections was used to gather data for the study. The first section gathered information on sociodemographic variables: age, gender, study locations, educational qualification, religion, designation, average monthly income and marital status. The second section consisted of health assessment measures for blood pressure, random blood sugar, height, weight, waist and hip circumference.

Ethical approval for the study was obtained from Ethics Committee of the University of KwaZulu-Natal, Durban, South Africa (BFC423/16) and the National Open University of Nigeria Health Research Ethics Review Committee (NHREC 04). An information sheet containing details of the survey was given to each participant. They were informed and assured that anonymity and confidentiality would be maintained. They were further assured that participation in this study was entirely voluntary, that they could withdraw from the study at any time, and that data from the survey would be used for scientific research purposes only.

Age was recorded in years; average monthly income less than N100 000 was recorded as low income, N101 000–N200,000 as middle income and above N200 000 as high income. The sociodemographic variables under study were age, gender, religion, location of staff, average monthly income, marital status, designation and highest educational level.

The health assessments section measured the cardiometabolic status (blood pressure, random blood sugar, height, weight, waist and hip circumference). Participants were made to rest in a sitting position for between 5 and 10 min before taking the blood pressure test. A digital sphygmomanometer was used to measure blood pressure twice, with an interval of 10 min, and an average of the two was recorded as the participant’s blood pressure. The blood pressure readings of the participants were described as normal systolic blood pressure when systolic measured below 120 mmHg, systolic prehypertension was between 120 mmHg and 139 mmHg, and systolic hypertension was < 140 mmHg and above. Normal diastolic blood pressure was diastolic measuring < 80 mmHg, diastolic prehypertension was blood pressure measuring between 80 mmHg and 89 mmHg, and diastolic hypertension was blood pressure measuring 90 and above [27, 34]. RBS was measured using glucometer and results were in mg/dL. RBS level of < 70–139 (3.9–7.7 mmol/l) and > 140 (7.8 mmol/l) and above were classified as normal and high RBS respectively. Height was measured in centimetres accurate to 0.1 cm using stadiometer. Weight was accurate to 0.1 kg using weighing scale. BMI was calculated by dividing body weight in kg by the square of the individual’s height in metres. BMI was classified according to the Centre for Disease Control [35] as ≤18 = underweight, 18.5–24.9 = normal or healthy weight, 25.0–29.9 = overweight and ≥ 30 = obesity. Waist circumference was measured at the midpoint between the lower margin of the least palpable rib and the top of the iliac crest and hip circumference was measured around the widest portion of the buttock, both with a tape measure accurate to 0.1 cm. WHR was calculated by dividing waist circumference by hip circumference. WHR classification based on WHO is healthy when ≤0.85 for women and ≤ 0.90 for men. Hence, we categorized WHR as healthy WHR when calculated at ≤ .85 and as risk WHR for > 0.86 [12].

### Data analysis

Data were analysed using Statistical Package for Social Science (IBM-SPSS version 25). Continuous variables were summarized as the mean ± standard deviation. Categorical data were summarized using frequency, percentage and cross-tabulation. Univariable and multivariable logistic regression was conducted to explore the association between sociodemographic variables and health status parameters of the participants. We initially performed a univariable logistic regression to estimate the association between each of the cardiometabolic-status and sociodemographic characteristics of the participants. Only the sociodemographic variables that were significant were put in the final models for each health status parameter. Hence, all the health status parameters were regrouped into binary (normal and high) as they were treated as the dependent variables. The blood pressure readings of the participants were therefore further classified as normal blood pressure when systolic measured < 140 and diastolic < 90, and as high blood pressure when systolic blood pressure ≥ 140 and diastolic ≥ 90. BMI was classified as normal BMI when calculated as < 18–24.9 and high BMI when the calculation was > 25.0. Four models were developed for each health status parameter and sociodemographic variable. Odds ratio (OR) was estimated with 95% confidence interval (CI). Cases with missing value/s were defined as no response, treated and expressed in frequency and percentage. However, in the regression analysis, missing cases were excluded and treated as missing. Statistical significance was determined at a *p*-value ≤ 0.05.

## Results

Table 1 shows the sociodemographic variables of the study participants. The largest age group among participants (45.5%) was the 30–39 years age group and mean age of participants was 40.13 ± 9.53 years, 55.7% were male, 87.1% were Christians, more than half (58.6%) had university education, 51.4% were senior non-academic staff, 79.3% were married, and 49.3%. were in the low monthly income category (less than N100 000).

**Table 1:** Sociodemographic variables of Nigerian university staff

Age	Frequency	Percentage
20–29	30	10.7
30–39	126	45.0
40–49	67	23.9
50–59	46	16.4
60–69	11	3.9
Gender		
Male	156	55.7
Female	124	44.3
Location of the study		
Headquarter	139	49.6
Study centres	141	50.4
Religion		
Christian	244	87.1
Islam	36	12.9
Marital status		
Married	207	73.9
Single	73	26.1
Educational qualification		
Primary school	16	5.7
Secondary school	56	20.0
College certificate	44	15.7
University certificate	164	58.6
Designation		
Academic Staff	30	10.7
Senior Non-academic Staff	144	51.4
Junior Non-academic Staff	106	35.4
No Response	7	2.5
Average monthly income		
Low income	138	49.3
Middle income	115	41.1
High income	27	9.6

**Fig. 2:**
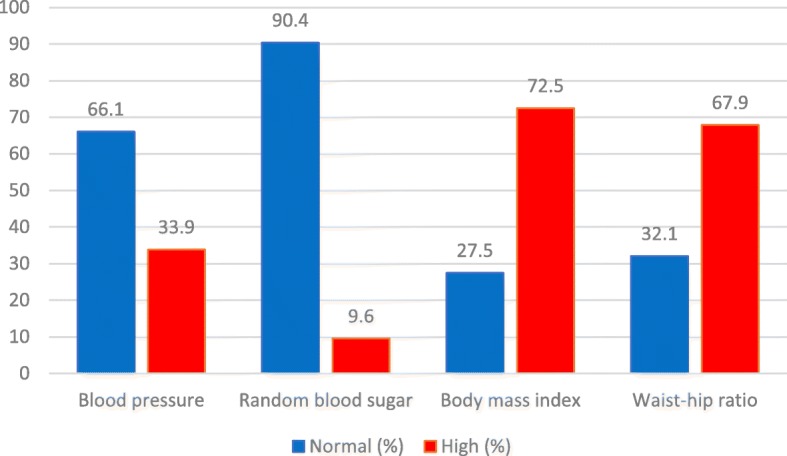
Health status parameters of Nigerian university staff (*N* = 280)

Figure 2 gives a summary of participants’ health status. Mean systolic blood pressure was 132.04 ± 19.20 with minimum value of 90 mmHg and maximum value of 195 mmHg. Mean diastolic blood pressure was 78.11 ± 0.81 with minimum value of 48 mmHg and maximum value of 116 mmHg. Mean BMI was 27.74 ± 5.22 and ranged between 16 and 52. Mean WHR was 0.88 ± 0.63 ranges between 0.72–1.24. Mean RBS was 98.65 ± 21.30 and ranged between 69 and 222 mg/dl. Prevalence of high blood pressure was 33.9%. Prevalence of high BMI was 72.5% and high WHR was 67.9%. Prevalence of high RBS was 9.6%.

To determine the predictor(s) of participants’ health status we conducted univariable logistic regression to analyse the relationship between health status parameters (body mass index, blood pressure, random blood sugar and waist-hip ratio) and predictors (age, gender, religion, highest educational level, designation and average monthly income), with crude odds ratios and 95% confidence intervals being estimated for each predictor. We further conducted multivariable logistic regression for all the predictors that were significantly associated with each health status in the univariable analysis. Four models were developed for each of the health status parameters.

The univariable logistic regression (Table 2) shows that age, gender, working location, marital status, highest educational level and average monthly income each had a statistical significant association with blood pressure of the participants. All the factors that had statistically significant association with blood pressure in the univariable analysis were used to build the multivariable logistic regression model. The final model reveals that age (aOR = 1.10; CI 95%: [1.05–1.14]) and gender (aOR = 0.5; CI 95%: [0.3–0.9]) were predictors of high blood pressure. Furthermore, employees in the institution study centres and married were more likely to develop high blood pressure, while highly educated employees with medium and high monthly income were less likely to have high blood pressure.

**Table 2:** Univariable and multivariable odds ratios (95% CI) for predictors of high blood pressure among university staff

Explanatory variables	Categories of explanatory variables	Univariable			Multivariable		
		Crude Odds ratio	Confidence interval	*p*-value	Adjusted odds ratio	Confidence interval	*p*-value
Age		1.096	1.064–1.129	0.000	1.098	1.054–1.144	0.000
Gender	Male	1			1		
	Female	0.629	0.379–1.045	0.073	0.501	0.275–0.912	0.024
Working locations	Headquarter	1			1		
	Study centres	2.355	1.413–3.921	0.001	1.707	0.936–3.113	0.081
Religion	Christian	1					
	Islam	0.838	0.393–1.786	0.647			
Marital status	Single	1			1	1	
	Married	3.028	1.563–5.865	0.001	1.386	0.652–2.946	0.396
Highest education level	Primary	1			1		
	Secondary	0.161	0.041–0.629	0.009	0.562	0.121–2.600	0.461
	Colleges	0.119	0.029–0.485	0.003	0.612	0.122–3.078	0.551
	University	0.085	0.023–0.311	0.000	0.483	0.095–2.454	0.381
Designation	No response	3.474	0.369–32.743	0.277			
	Academic	1.927	0.224–16.556	0.550			
	Senior non-academic	5.647	0.656–48.643	0.115			
	Junior non-academic	1					
Average monthly income	Low	1			1		
	Medium	0.407	0.233–0.711	0.002	0.497	0.217–1.136	0.098
	High	1.577	0.689–3.609	0.281	0.596	0.170–2.091	0.419

Age, gender, religion, marital status, designation and monthly income had statistically significant association with high BMI. The multivariable logistic regression results (Table 3) further demonstrated that gender (aOR = 2.3, CI 95%: [1.2–4.2]) and religion (aOR = 0.3, CI 95%: [0.2–0.7]) were the factors that statistically significantly associate with high BMI. Older (aOR = 1.0, CI 95%: [0.1–1.8]) and married participants (aOR = 1.90, CI 95%: [0.96–3.78]) were more likely to have high BMI but not statistically significant. Muslim participants were less likely to have high BMI (aOR = 0.3, CI 95%: [0.15–0.72]) and is statistically significant to high BMI.

**Table 3:** Univariable and multivariable odds ratios (95% CI) for predictors of high body mass index among university staff

Explanatory variables	Categories of explanatory variables	Univariable			Multivariable		
		Crude odds ratio	Confidence interval	*p*-value	Adjusted odds ratio	Confidence interval	*p*-value
Age		1.054	1.021–1.087	0.001	1.037	0.997–1.079	0.071
Gender	Male	1			1		
	Female	2.525	1.434–4.446	0.001	2.281	1.252–4.156	0.007
Working locations	Headquarter	1					
	Study centre	0.738	0.436–1.250	0.259			
Religion	Christian	1			1		
	Islam	0.364	0.178–0.746	0.006	0.330	0.151–0.722	0.006
Marital status	Single	1			1		
	Married	0.458	0.259–0.809	0.007	1.903	0.957–3.784	0.067
Highest education level	Primary	1					
	Secondary	1.202	0.391–3.698	0.748			
	Colleges	2.644	0.786–8.900	0.116			
	University	2.579	0.900–7.386	0.078			
Designation	No response	3.429	0.397–29.618	0.263	5.115	0.504–51.940	0.168
	Academic	3.714	1.200–11.493	0.023	3.198	0.710–14.405	0.130
	Senior non-academic	1.714	0.982–2.991	0.058	1.8431	0.765–4.493	0.173
	Junior non-academic	1			1		
Average monthly income	Low	1			1		
	Medium	1.605	0.924–2.789	0.093	0.934	0.391–2.229	0.878
	High	6.456	1.466–28.437	0.014	1.522	0.245–9.437	0.652

Table 4 give a summary of the predictors of high waist-hip ratio. Gender (OR = 0.3, CI 95%: [0.2–0.4]) is the only factor that had statistical significant association with high waist-hip ratio among the participants.

**Table 4:** Univariable and multivariable odds ratios (95% CI) for predictors of high waist-hip ratio among university staff

Explanatory variables	Categories of explanatory variables	Univariable			Multivariable		
		Crude odds ratio	Confidence interval	*p*-value	Adjusted odds ratio	Confidence interval	*p*-value
Age		1.027	0.999–1.056	0.056			
Gender	Male	1					
	Female	0.273	0.161–0.463	0.000			
Working locations	Headquarter	1					
	Study centre	0.957	0.579–1.580	0.862			
Religion	Christian	1					
	Islam	1.268	0.583–2.758	0.549			
Marital status	Single	1					
	Married	0585	0.336–1.019	0.058			
Highest education level	Primary	1					
	Secondary	0.449	0.114–1.772	0.253			
	Colleges	0.692	0.166–2.890	0.614			
	University	0.433	0.119–1.583	0.208			
Designation	No response	1					
	Academic	3.281	0.380–28.359	0.280			
	Senior non-academic	0.820	0.355–1.898	0.643			
	Junior non-academic	1.328	0.769–2.294	0.309			
Average monthly income	Low	1					
	Medium	0.948	0.559–1.609	0.844			
	High	1.112	0.452–2.735	0.818			

Table 5, giving the final model of health status shows that only age (OR = 1. 04, CI 95%: [1.00–1.09]) had a statistically significant relationship with RBS.

**Table 5:** Univariable and multivariable odds ratios (95% CI) for predictors of high random blood sugar among university staff

Explanatory variables	Categories of explanatory variables	Univariable			Multivariable		
		Crude odds ratio	Confidence interval	*p-*value	Adjusted odds ratio	Confidence interval	*p*-value
Age		1.044	1.003–1.086	0.037			
Gender	Male	1					
	Female	1.087	0.484–2.443	0.840			
Working locations	Headquarter	1					
	Study centre	1.385	0.613–3.133	0.434			
Religion	Christian	1					
	Muslim	1.261	0.408–3.897	0.687			
Marital status	Single	1					
	Married	2.051	0.682–6.167	0.201			
Highest education level	Primary	1					
	Secondary	0.231	0.050–1.057	0.059			
	Colleges	0.220	0.043–1.120	0.068			
	University	0.302	0.087–1.054	0.060			
Designation	No response	1.667	0.180–	0.653			
	Academic	0.714	15.425	0.678			
	Senior non- academic	1.077	0.146–3.502	0.869			
		1	0.447–2.595				
	Junior non-academic						
Average monthly income	Low	1					
	Medium	1.221	0.509–2.929	0.654			
	High	2.008	0.588–6.853	0.266			

## Discussion

Our study assessed the health status of the university staff in Nigeria and its predictors. The study showed that quite a number of our study participants had elevated health status parameters. Age and gender were predictors of high blood pressure and gender and religion were predictors of BMI. Furthermore, worker’s location, marital status, monthly income, designation, highest education, religion, gender and age had statistically significant relationship with one or more measures of the health status As mentioned in the background of the study, overweight and obesity are linked to the development of CVDs, diabetes [2] and cancer [15].

The findings of our study are similar to another study conducted among university staff in south-west Nigeria where 75% of the study population had two or more cardiovascular risk factors [31]. Also, a study conducted among the market population in Enugu, Southeast Nigeria reported similar findings in relation to blood pressure, WHR and BMI [28]. There were high prevalence rate of hypertension (33%) and high BMI (72.5%) in this current study compared to the study conducted among a semi-urban community in South South Nigeria, that reported prevalence rates of hypertension and high BMI to be to be 15% and 51.2% respectively [36]. The prevalence rates of both systolic and diastolic hypertension reported is higher than that reported among rural dwellers in South West Nigeria [37]. Despite the differences in the prevalence rate of hypertension in term of geographical location, evidence from two reviews conducted on the prevalence of hypertension showed that there is a steady increase in the prevalence of hypertension in both rural and urban areas [3, 26]. There is wide variation between the prevalence rates of overweight and obesity found in this current study compared to review reports on BMI between 2001 and 2012 which were between 28.4 and 57.3% [38].

This study findings reveal that religion had statistical significant impact on BMI and Muslim participants were less likely to have high BMI. Similar results were obtained among England and Australia population where religious denomination [39] and religion affiliation [40] had statistical significant relationship with high BMI. Lycett (2015), further reported that Christian participants had higher BMI values compared to participants with other religion faith [40]. The explanation to this current study results might be that periodic fasting of Islamic religion could have contributed to the lower BMI values. According to Levitt, type 2 diabetes accounted for 90.0% of all cases of diabetes in sub-Sahara Africa [41]. Another study reported 2.5% prevalence rate of impaired fasting blood sugar among the semi-urban population in Northern Nigeria [42]. Our study reported that 9.5% of the population had an abnormal RBS value.

The high prevalence of overweight (41.4%), obesity (31.8%) and risk WHR (67.9%) is worrisome because of their relationship with CVDs and diabetes [1, 7, 28, 41, 43]. A recent document released by the American Heart Association [44] defines elevated blood pressure as systolic of 120 and above and diastolic of 80 and above [45]. This definition will increase the number of our study participants with elevated blood pressure to 48.2%. Our study reported that females are less likely to have high blood pressure compared to their male counterpart. The possible explanation to this findings is that premenopausal women are less likely to have cardiovascular disorders compared to their men counterpart [46] . Majority of our participants were within the age range of 20–49 years which further buttress the fact that abundance oestrogen hormone present in women prior to menopause protect women from aldosterone-induced hypertension [46]. Furthermore, male sex and hypertension were among predictors of coronary heart diseases [18]. The findings on the predictors of health status are critical as all the sociodemographic variables contributed to the development of one or more of the risk factors for cardiometabolic disorders. WHR is suggested to be the best predictor of CVD risk [12], and it is associated with increased risk to diabetes, hypertension among all ethnic groups [43]. In men, high WHR increased the risk of CVD incidence, morbidity and mortality compared to BMI, and BMI was associated with increased risk of CVDs in women [12, 43].

The findings from this study are therefore crucially important in guiding intervention programmes for the university staff and workers generally. Surveillance is crucial in the control and prevention of NCDs [5, 9, 47], especially since some of the variables that influence some of the risk factors like age and gender [27, 28] cannot be controlled. Likewise, there are factors such as socioeconomic status which could in theory be controlled except that the means are not available (e.g. low income, as reported by roughly half our study participants). Literature revealed colossal amounts spent in the management of NCDs [1, 2, 47, 48], making it all the more essential to develop an intervention for control and prevention of NCDs that is not capital intensive and that will include all of the university staff population.

One of the limitations of this study was that the testing for RBS was not good enough to predict prevalence of impaired glucose tolerance or diabetes among the study population. The difficulty was that participants were often too busy to attend following a two-hour post-feeding interval for the RBS testing so that the post-feeding interval was in most cases much longer. Hence it would be advisable to perform fasting blood sugar examination to evaluate the prevalence of diabetes among the study population.

## Conclusion

In conclusion the health status of the study population is borderline. The prevalence rates of high blood pressure and random blood sugar; overweight, obesity and risk WHR are on the increase compared to previous studies, with lifestyle behaviour of university staff [24] a possible explanation for this finding. The findings of this study are useful in the planning of intervention programmes for the control of diabetes and hypertension that usually co-exist and share common risk factors like overweight and obesity. With the findings of this study, interventions that can increase physical activities and healthy nutritional lifestyle behaviour should be developed for workers so as to achieve and maintain healthy weight. Health responsibility is key in the control and prevention of NCD, therefore, workers should be empowered with adequate information that can assist them take control of their health. Finally, nurses most especially community/public health nurse practitioners working with institutions should develop interventions that includes awareness campaigns, continuous screening and surveillance to prevent NCDs and their associated disabilities.

## Additional file

Additional file 1: Health status questionnaire (DOCX 14 kb)

## Abbreviations

BMI: Body mass index
NCDs: Non-communicable diseases
RBS: Random blood sugar
WHR: Waist-hip ratio
