# Supplementary material for: Evaluation of health status and its predictor among university staff in Nigeria
Source: BMC Cardiovasc Disord. 2018 Sep 20;18:183. doi: 10.1186/s12872-018-0918-x (PMC6148776; doi:10.1186/s12872-018-0918-x)
Supplement: Supplementary file 1 — Health status questionnaire (DOCX 14 kb) [file 12872_2018_918_MOESM1_ESM.docx]

Health status questionnaire of University staff

**SECTION A**

**Personal Attributes and Socio-Demographic Characteristics**

**Instruction**: Please write or tick as appropriate.

1. Age in years (Please specify) ______________________________________
2. Gender: (a) Male ( ) (b) Female ( )
3. Study Location (a) Headquarters ( ) (b) Ibadan SC ( ) (c) Benin SC ( ) (d) Enugu SC ( ) (e) Kaduna SC ( ) (f) Gombe SC ( ) (g) Lokoja SC ( )
4. Religion (a) Christian ( ) (b) Islam ( ) (c) traditional ( ) (d) Others (specify) ____________ Please, specify denomination __________________________________
5. Highest educational level: (a) Primary school ( ) (b) Secondary School ( ) College Certificate ( ) University Certificate ( )
6. Designation (a) Academic staff ( ) (b) Senior Non-academic Staff ( ) (c) Junior Non-academic Staff ( )
7. Marital status: (a) Married ( ) (b) Divorced ( ) (c) Widowed ( ) (d) Separated ( )

(e) Single ( )

1. Type of marriage: (a) Monogamy ( ) (b) Polygamy ( )
2. Numbers of children that you have (Please specify) ______________________
3. Average income in a month: (a) Less than N50,000 ( ) (b) Between 51,000 and 100,000 ( ) (c) Between 101,000 and 150, 000 ( ) (d) Between 151 and 200,000 ( ) (e) 200,000 and above ( )

**SECTION B**: INDICATORS TO ASSESS THE HEALTH STATUS OF STAFF

- Blood pressure - Random blood sugar
- Weight - Height
- Body mass index (BMI) - Waist circumference
- Hip circumference
